# Stress-relieving plant growth-promoting bacterial co-inoculation enhances nodulation and nitrogen uptake in black gram under nitrogen-free saline conditions

**DOI:** 10.3389/fmicb.2024.1516748

**Published:** 2025-01-03

**Authors:** Praveen Kumar Tiwari, Anchal Kumar Srivastava, Rachana Singh, Alok Kumar Srivastava

**Affiliations:** ^1^National Bureau of Agriculturally Important Microorganism, Mau, India; ^2^Amity Institute of Biotechnology, Amity University Uttar Pradesh, Lucknow Campus, Lucknow, India

**Keywords:** salt stress, phytohormones, co-inoculation, chlorophyll content, nutrient uptake, nodulation

## Abstract

Non-halophytic plants are highly susceptible to salt stress, but numerous studies have shown that halo-tolerant microorganisms can alleviate this stress by producing phytohormones and enhancing nutrient availability. This study aimed to identify and evaluate native microbial communities from salt-affected regions to boost black gram (*Vigna mungo*) resilience against salinity, while improving plant growth, nitrogen uptake, and nodulation in saline environments. Six soil samples were collected from a salt-affected region in eastern Uttar Pradesh, revealing high electrical conductivity (EC) and pH, along with low nutrient availability. A total of 72 bacterial strains were isolated from soil and 28 from black gram (*Vigna mungo*) root nodules, with 32 of the soil bacteria tolerating up to 10% NaCl. These bacteria were characterized through taxonomic and biochemical tests. Cross-compatibility analysis showed two rhizobia strains were highly compatible with five salt-tolerant bacteria. These strains exhibited significant plant growth-promoting traits, including phosphate, potassium, and zinc solubilization, as well as ACC deaminase, IAA, siderophore, and EPS production. Strain *Paenibacillus* sp. SPR11 showed the strongest overall performance. Genetic diversity was assessed using BOX-PCR and ERIC-PCR, and strains were identified through 16S rRNA gene sequencing. In a seed germination study under saline conditions (200 mM and 300 mM), co-inoculation with *Bradyrhizobium yuanmingense* PR3 and *Paenibacillus* sp. SPR11 resulted in a significant enhancement in seed germination (40%), root growth (84.45%), and shoot growth (90.15%) compared to single inoculation of *B. yuanmingense* PR3. Under greenhouse conditions in Leonard jars, co-inoculation with strains PR3 and SPR11 significantly enhanced shoot and root length, fresh and dry biomass, nodule count, and nodule fresh and dry weight. Chlorophyll content, nutrient uptake, and crude protein levels increased, while proline content decreased compared to single inoculation and uninoculated seeds. Our best understanding leads us to believe that this is the very first report of utilizing co-inoculation of salt-tolerant *Paenibacillus* sp. SPR11 and *B. yuanmingense* PR3, demonstrating their promising potential to alleviate salt stress and enhance growth, root architecture, nitrogen uptake, and nodule formation in black gram under nitrogen free saline conditions.

## Graphical abstract

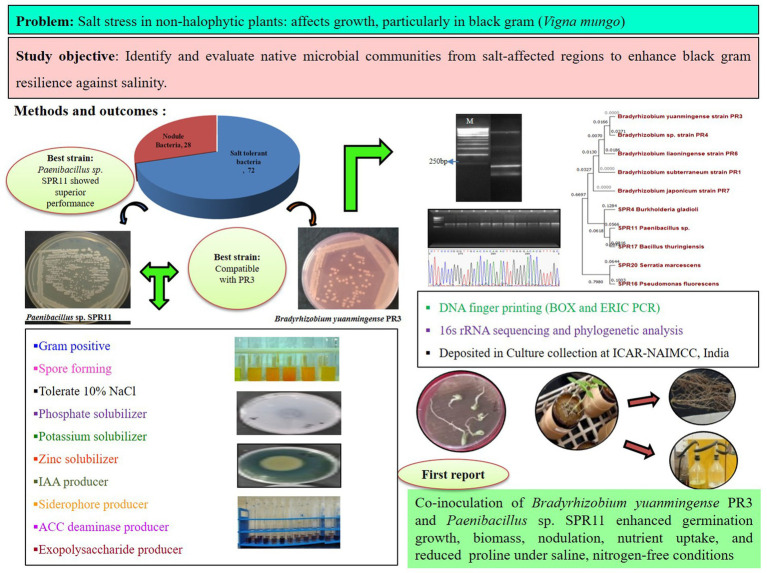

## 1 Introduction

Soil salinity represents a significant challenge to agriculture worldwide, affecting approximately 1,125 million hectares globally, including 6.73 million hectares in India (Mishra et al., 2023). In India, around 2.5 million hectares are impacted by soil salinity and alkalinity, with approximately 1.2 million hectares located in Uttar Pradesh. This includes the districts of the Ganga-Yamuna and Ganga-Gomti doab regions, particularly in Eastern U.P., where soil degradation is most pronounced (Priyadarshi and Yadav, 2023). This problem hampers crop productivity and threatens food security, particularly in areas with little rainfall, high temperatures, poor irrigation, and heavy use of chemical fertilizers (Raza et al., 2019; Kumar and Sharma, 2020). The black gram is one of the important pulse crop crops, severely affected by the soil salinity and makes up to 11% of India’s total pulse production, which was 25.46 million tons in 2020–2021 (Agricultural Statistics Division, Ministry of Agriculture and Farmers’ Welfare, India, 2022). Though India is the major producer and consumer of black gram, the high salinity in the soil reduces its yield and makes it hard to meet the local demand. Hence, it is crucial to find an economical and sustainable solution to deal with salinity to improve black gram production and ensure food security in India (Atta et al., 2023). Nitrogen fixation in leguminous crops is significantly impaired when exposed to a salinity level of up to 6 dS m^−1^. The elevated levels of Na^+^ and Cl^−^ in the roots and shoots can lead to disruptions in osmotic balance, adversely affecting the plant’s metabolic pathways, leading to lower crop yields and reduced levels of leghemoglobin in the nodules and overall health (Goyal and Habtewold, 2023; Comba et al., 1998; Ali et al., 2023; Guinel, 2015; Reid et al., 2017). Nitrogen, a vital element for the development of all plant structures, serves as a critical component of chlorophyll, enzymes, and proteins, and is required in larger quantities than most other plant nutrients (Sabagh et al., 2020). Leguminous crops naturally meet their nitrogen requirements through a symbiotic relationship with nitrogen-fixing bacteria (Bhattacharjee et al., 2008; Castro et al., 2016; Babalola et al., 2017; Kawaka, 2022). Despite this inherent advantage, chemical fertilizer usage remains high in India. In the 2022–2023 period, India consumed 35.73 million metric tons of urea and 10.53 million metric tons of DAP, according to sales data from DBT India (Song et al., 2019). Research suggests that co-inoculating legumes with salt-tolerant soil bacteria, in combination with rhizobia, is a sustainable approach to enhance nitrogen availability through nitrogen fixation and improve the productivity of leguminous crops in saline soil. This method proves more effective than using a single rhizobium (Ahmad et al., 2011; Nitawaki et al., 2021). The nature-friendly bacteria can contribute to improved plant growth through several direct mechanisms viz. solubilization of phosphate, production of indole acetic acid (IAA), exopolysaccharides (EPS), siderophores, ammonia, ACC deaminase, antioxidants as well as fixation of nitrogen. They also enhance growth indirectly by providing antagonistic effects and inducing systemic resistance (Upadhyay et al., 2011; Chandran et al., 2021). PGP bacteria enhance the plant growth by up-regulating the production of Na^+^/K^+^ ion channel proteins, hence facilitating ionic balancing and enhancing plant resilience under saltwater conditions (Egamberdieva et al., 2019). They also help to protect plants from diseases in both normal and salty conditions (Mahgoub et al., 2021). Certain PGP bacteria such as *Bacillus, Enterobacter* sp.*, Pseudomonas* sp., *Arthrobacter* sp., *Azotobacter* sp., *Azospirillum* sp.*, Serratia* sp. and Rhizobia, have displayed promising responses in helping the plants to tolerate salt, superior by reducing the adverse consequences of salinity and alkalinity (Hussain et al., 2020; John et al., 2023). Furthermore, the study has shown that *Paenibacillus* strains are capable of surviving salt stress at a level of up to 15% NaCl and possess advantageous characteristics such as solubilization of phosphorus, production of siderophore and IAA (Gopal and Anandham, 2020). Combining PGPR strains that produce ACC deaminase with rhizobial strains can significantly improve salt tolerance, growth, and nodulation in various crops under saline conditions (Ahmad et al., 2011; Chandran et al., 2021; Win et al., 2023). Therefore, the research aimed to evaluate the efficacy of “salt-tolerant plant growth-promoting strains” derived from saline soils in enhancing plant growth, nitrogen uptake and nodulation under salt stress. The most effective strain, *Paenibacillus* sp. SPR11 was chosen based on salt- tolerance, biochemical potential, *in vitro* plant growth-promoting (PGP) characteristics, compatibility, DNA fingerprinting, and 16S rRNA gene sequencing analysis. A seed germination test and Leneord jar experiment were then conducted to assess how co-inoculating SPR11 and PR3 affect plant growth and nodulation in black gram underneath nitrogen-free saline conditions.

## 2 Materials and methods

### 2.1 Sampling of soil and root nodules

From eastern Uttar Pradesh, a total of six soil samples were collected from saline soil of Jaunpur (latitude 25.967100, longitude 82.682800), Azamgarh (latitude 26.055200, longitude 82.681500), and Mau district (latitude 25.972970, longitude 83.574640). For grid sampling, collected composite 10 subsamples for each side of salt affected area of each location (Supplementary Table S1). These samples were taken from the uppermost part of the soil which spans 15 cm. In addition to this, black gram root nodules were collected from Jaunpur and Mau (ICAR-CSSRI/Bharuch/Technical Manual/2017/07) (Table 1). The soils were sieved by 2 mm mesh, shade-dried, and then stored at 4°C for further analysis.

**Table 1:** Analysis of physicochemical properties of soil samples from different locations in eastern Uttar Pradesh.

Soil code	pH	EC (dS m^−1^)	SOC (%)	N (kg ha^−1^)	P (kg ha^−1^)	K (kg ha^−1^)
JN1	9.310	9.61	1.11 ± 1.38^a^	276.73 ± 1.78^a^	83.67 ± 1.31^bc^	138.88 ± 2.41^a^
JN2	8.510	8.62	0.63 ± 1.31^abc^	166.82 ± 1.63^cd^	79.30 ± 1.12^c^	137.76 ± 2.69^ab^
MU1	8.72	7.85	0.84 ± 2.21^b^	218.52 ± 1.48^b^	101.15 ± 1.08^ab^	123.98 ± 3.23^bc^
MU2	8.1	9.26	0.49 ± 2.48^c^	148.40 ± 1.17^d^	99.70 ± 1.20^abc^	119.84 ± 1.99^bc^
AZ1	8.15	7.79	0.46 ± 1.38^cd^	189.16 ± 2.05^bc^	114.27 ± 0.98^a^	105.61 ± 2.98^d^
AZ2	8.93	8.93	0.24 ± 1.88^e^	103.22 ± 1.68^e^	114.27 ± 1.10^a^	99.68 ± 2.62^e^

Data are presented as means with standard deviations (*n* = 3). Different letters indicate significant differences between locations, as determined by Duncan’s multiple range test (*p* ≤ 0.05).

### 2.2 Physio-chemical analysis of soil

A soil water solution was prepared by mixing 10 g of soil with 25 mL of distilled water in a 1:2.5 ratio. The pH of the resulting suspension was measured using a pH meter (Eutech Instruments, India) following the method described by Thomas (1996) while the same suspension was used to measure the electrical conductivity with EC meter (Labman Instrument India) with readings given in dS m^−1^ (Rhoades et al., 1999). Soil organic carbon content was measured with a rapid chromic titration method developed by Walkley and Black (1934). The semi-auto nitrogen analyzer was used to find the available nitrogen of the soil (Subbiah and Asija, 1956). Olsen’s et al. (1954) method was used to find out the available phosphorus of the soil. Flame photometry was the method to measure available potassium in soil by Jackson (1973).

### 2.3 Isolation and screening of salt-tolerant bacteria

Soil bacteria were isolated using the serial dilution method, up to a dilution factor of 10^−5^, by spreading the diluted samples onto various media, including Nutrient Agar, King’s B, R2A, Jensen agar, and Soybean Casein Agar. The plates were incubated at 30°C for 3–5 days. To isolate nodule bacteria, black gram nodules were thoroughly washed with running tap water and carefully detached from the roots. Surface sterilization was performed by 70% ethanol for 1 min, followed by 2% sodium hypo-chloride for 2 min (Somasegaran and Hoben, 1992). To complete the sterilization process, the nodules were rinsed five times with sterile water and then crushed in 1 mL of sterilized Milli-Q water using a mortar and pestle. Serial dilution of resultant nodules suspension to 10^−3^ to 10^−5^ followed by spreading of 100 μL aliquots of each dilution onto Yeast extract mannitol agar medium enriched with 0.0025% (w/v) Congo red. After 3 to 5 days of incubation at 30°C, the growth of nodule bacteria was assessed (Somasegaran and Hoben, 2012). To evaluate the salt tolerance of the bacterial strains, nutrient agar plates were prepared with NaCl concentrations of 2, 4, 6, 8, and 10%. These plates were incubated at 30°C for 3–5 days, as described by Robinson et al. (2015) and Chaudhary et al. (2019).

### 2.4 Morphological studies and biochemical test

The bacterial isolates were characterized microscopically using Gram staining and endospore staining, which facilitated the observation of colony morphology, including various shapes and motility, under both compound and stereomicroscopes. Additionally, several biochemical tests were conducted, including the methyl red test (MR), Voges–Proskauer test (VP), nitrate reduction test (NR), citrate utilization test (CU), and amylase production. The hydrolysis of carbohydrates such as mannitol, dextrose, lactose, and sucrose was also assessed (Ventosa et al., 1998). Presumptive tests for the nodule bacteria were conducted on yeast extract mannitol agar plates containing Congo red. The nodule bacteria were streaked on peptone glucose agar and ketolactose agar, and then incubated at 30°C for 3 to 5 days. Rhizobial isolates were expected to show little to no growth on peptone glucose agar. When ketolactose agar plates were treated with Benedict’s solution, contaminants produced a yellow zone around their growth due to the utilization of 3-ketolactose, whereas rhizobial colonies did not exhibit this yellow zone (Legesse, 2016). Additionally, Hoffer’s (1935) alkaline test was performed to differentiate between *Agrobacterium* and rhizobia as it is based on their ability to grow at higher pH levels. *Agrobacterium* can grow in an alkaline medium with a pH 11, whereas rhizobium cannot grow on this pH.

### 2.5 *In vitro* study of compatibility test

In this study, the compatibility of the various strains that were evaluated using a cross-streak assay on a 1:1 mixture of nutrient agar and yeast extract mannitol agar with minor modification (Santiago et al., 2017). Initially, rhizobia strains were streaked onto mixed agar plates and incubated at a temperature of 30°C for 48 h. Subsequently, the salt-tolerant bacterial strain was streaked vertically against the nodule bacteria. Bacterial growth on the plates was monitored for 24 h at 30°C. Mixing of growth from both strains was seen as compatible if the counter bacterial strain’s development being suppressed by the already streaked bacterial strain was considered as incompatible.

### 2.6 *In vitro* study of plant growth-promoting traits

#### 2.6.1 Indole acetic acid production assay

Bacterial cultures were inoculated in 5 mL of nutrient broth (NB) supplemented with 0.2% L-tryptophan and 2% NaCl (w/v). The cultures were incubated at 30°C on a shaker at 150 rpm for 48 h (Khoso et al., 2023). After incubation, the cultures were centrifuged, and an equal volume of Salkowski reagent was added to the culture supernatant and incubated at room temperature for 30 min. The development of a pink color indicated the production of indole-3-acetic acid (IAA). The optical density was measured using a spectrophotometer at 530 nm, and the IAA concentrations were compared to a standard IAA solution (Bric et al., 1991).

#### 2.6.2 Siderophore production assay

Siderophore production was assessed using spot inoculation on chrome azurole sulfonate (CAS) medium supplemented with 2% NaCl (Schwyn and Neilands, 1987). The plates were incubated at 30°C for 48 h, and the presence of a yellow to orange halo around the bacterial colonies was considered indicative of positive siderophore production.

#### 2.6.3 ACC deaminase activity test

The bacterial cultures were inoculated in DF broth supplemented with 3 mM ACC with 2% NaCl (w/v) as described by Dworkin and Foster (1958). The cultures were inoculated and inoculated for 48 h of incubation on a rotary shaker at 30°C, and growth and turbidity were monitored to access ACC deaminase activity. Following incubation, the bacterial cells were centrifuged, harvested, and washed three times with Tris-HCl (pH-7.5) buffer then re-suspended in 1 mL 0.1 M Tris-HCl (pH- 8.5). To the cell suspension, 30 μL of toluene was added as per the methods of Penrose and Glick (2003). An aliquot of cell suspension was used to quantify α-ketobutyrateto and ACC deaminase activity was measured by determining the absorbance at 540 nm. The absorbance values were compared to a standard curve of varying concentrations of α-ketobutyrate to calculate the level of ACC deaminase activity.

#### 2.6.4 Phosphate solubilization activity

Phosphorus solubilization was evaluated using Pikovskaya agar media supplemented with 2%NaCl (w/v). Bacterial cultures were spotted on the plates and incubated at 30°C for 48 h. According to Tank and Saraf (2010) the formation of a zone of clearance around the bacterial colony indicated a positive result for phosphate solubilization. The solubilization index (SI) for phosphorus was determined through qualitative analysis using the following formula


$$\text{Solubilization index}=\frac{\text{Colony diameter}-\text{Halo zone diameter}}{\text{Colony diameter}}$$

#### 2.6.5 Potassium solubilization

Potassium solubilization studies were carried out using Aleksandrov solid medium supplemented with 2% NaCl (w/v). A 20 μL of culture broth were inoculated in to medium and incubated at 30°C for 48 h. The presence of a clear zone around the bacterial colonies indicated that potassium had been dissolved by the microorganisms (Meena et al., 2016). The solubilization index (SI) for potassium was determined through qualitative analysis using the following formula


$$\text{Solubilization index}=\frac{\text{Colony diameter}-\text{Halo zone diameter}}{\text{Colony diameter}}$$

#### 2.6.6 Zinc solubilization

Zinc solubilization was tested using insoluble zinc compounds such as zinc oxide (ZnO) and basal medium with 2% NaCl (w/v). The plates were spotted with bacterial broths and incubated at 30°C for 48 h. The formation of a halo zone around the bacterial culture spot showed identified as positive zinc solubilizers (Sharma et al., 2012). The solubilization index (SI) for zinc was determined through qualitative analysis using the following formula


$$\text{Solubilization index}=\frac{\text{Colony diameter}-\text{Halo zone diameter}}{\text{Colony diameter}}$$

#### 2.6.7 Synthesis of exopolysaccharide

EPS production was measured following the method described by Qurashi and Sabri (2011). The 100 mL of 48-h grown broth culture supplemented with 2% NaCl (w/v) was centrifuged at 10,000 rpm for 20 min in 4°C. To aid in the separation of EPS from the supernatant, prechilled acetone was added three times. The precipitated EPS was filtered after 2 days by the Whatman filter paper. The filters, containing the EPS, were dried overnight at 58° and the weighed of filter paper was recorded. The weight gain of the filter paper showed was EPS production.

### 2.7 DNA fingerprinting using ERIC and BOX PCR

The fingerprints of the bacterial genome were established to determine the phylogenetic relatedness of the different bacterial isolates as described by Li et al. (2017). ERIC-PCR and the BOX-PCR technique were done to evaluate the similarities between the 10 potential bacterial strains using clustering analysis. The Nucleo-pore gDNA Fungal/Bacterial Mini Kit (Genetix Biotech Asia Pvt. Ltd.) was used to isolate the genomic DNA following the manufacturer’s instructions. ERIC-PCR was performed using the primer 1R (ATGTAAGCTCCTGGGGATTCAC) and the 2F (AAGTAAGTGACTGGGGTGAGC) along with BOX-PCR primer 1 (CTACGGCAAGGCGACGCCTGACG) for DNA fingerprinting. The polymerase chain reaction (PCR) was carried out for 35 cycles under the following conditions: initial denaturation at 95°C for 7 min, denaturation at 94°C for 1 min, annealing at 52°C for 1 min, and extension at 65°C (ERIC) and 72°C for BOX. A final extension step was conducted at 65°C for ERIC and 72°C for BOX for 15 min. The amplified products using 2.0% agarose gel electrophoresis with a 100 bp DNA marker were visualized in a UV transilluminator (Bio-Rad). The Jaccard coefficient was used to compute the genetic similarity recorded as binary matrix for presence/absence. The UPGMA technique was used to create a dendrogram, while DARWin software (version 6.0.21) was used for statistical analysis.

### 2.8 Molecular identification through 16S rRNA gene sequencing

Five salt tolerant bacterial isolates (SPR4, SPR11, SPR16, SPR17, and SPR20) and five root nodule bacteria (PR1, PR3, PR4, PR6, and PR7) were selected for 16S rRNA sequencing based on their compatibility and beneficial Plant growth activities. The universal primer pA (5′AGAGTTTGATCCTGGCTCAG3′) and pH (3′AAGGAGGTGATCCAGCCGCA5′) (Edwards et al., 1989) were used for amplification. The amplified products of 16S rRNA were loaded along with 1 kb DNA molecular weight marker in 1.2% agarose gel containing 0.5 mg/mL ethidium bromide placed in running 1X TAE buffer for the electrophoresis. Following electrophoresis, the 16S rRNA amplified products were purified using the Sure Extract PCR/Gel Extraction Kit. The quality and quantity purified 16S rRNA amplified products were quantified using a Nanodrop spectrophotometer (DeNovix). 16S rRNA purified gene products were sequenced at the ICAR-NBAIM Genome Sequencing Lab, Mau, India, using an Applied Biosystems ABI 3130XL capillary genetic analyzer (United States). The sequencing data were analyzed using BLAST algorithm in the EZbiocloud and NCBI databases. The resulting 16S rRNA gene sequences were deposited in the NCBI GenBank sequence database. A phylogenetic tree was constructed using the MEGA 11 software, employing of neighbor-joining (NJ) phylogenetic dendrogram and maximum parsimony (MP) method with 1,000 bootstrapped replicates. The identified cultures were deposited at the ICAR-National Agriculturally Important Microbial Culture Collection (NAIMCC) in Mau, India and accession numbers were obtained.

### 2.9 Inoculums preparation

Based on the compatibility analysis, two rhizobial strains and five salt-tolerant bacteria were used in various combinations for co-inoculation. These combinations were PR3, PR4, PR3 × SPR11, PR3 × SPR16, PR4 × SPR4, PR4 × SPR17, and PR3 × SPR20. The inoculums of each strain was prepared of five-day-old cultures of rhizobial strains and 24-h-old cultures of salt-tolerant plant growth-promoting bacteria (PGPB) strains at 200 rpm in a rotary shaker until the cultures reached the logarithmic phase with a turbidity of around 10^9^ CFU mL^−1^. The broth cultures were washed and resuspended in phosphate buffer (0.1 M, pH 7.0) for inoculation of seed for different treatments. Thereafter, the seeds were air-dried for 1 h in the shade before sowing.

### 2.10 Co-inoculation effect on seed germination of black gram under salt stress

The PGP bacterial strains, previously identified as most compatible in different combinations, were tested for their ability to enhance seed germination under saline stress (200 mM and 300 mM NaCl, w/v) with positive control as well as without NaCl and bacterial culture served as negative control. The Black gram seeds (PU-6 variety) were procured from ICAR-IISS, Mau Nath Bhanjan, Uttar Pradesh. Prior to the experiment, the seeds were surface sterilized by immersing them in 70% ethanol for 3 min and subsequently 2% sodium hypochlorite for 5 min, washed five times with autoclaved Milli-Q water to remove any residual chemicals. The seeds were then treated with bacterial broth cultures, prepared to achieve a population density of 10^8^–10^9^ CFU/mL, as described as earlier whereas control seeds sterile shocked Milli-Q water. The inoculated seeds were placed in sterilized Petri dishes containing two layers of filter paper, which were saturated with nitrogen-free nutrient solution (Supplementary Table S2). The dishes were exposed to both saline (200 mM and 300 mM NaCl) and non-saline conditions. Each treatment was performed with four replicates, each containing five seeds. The Petri dishes were incubated at 30°C with a 10-h light and 14-h dark cycle. Growth parameters, including shoot length, radicle length, plumule length, root length, and germination percentage, were measured at various time intervals, following the methodology outlined by Noreen and Ashraf (2007).

### 2.11 Evaluation of microbial co-inoculation on growth and nodulation of black gram

For the greenhouse assay, following treatments (PR3, SPR11, PR3 × SPR11) were selected based on the highest germination rates observed in high-salinity-exposed black gram seeds with un-treated positive control. The Leonard jar method was employed for this experiment. In this method, sand was sieved through a 2 mm filter and sterilized via autoclaving. The sterilized sand was then filled into glass bottle jars, which were arranged in a completely randomized design, with five replications for each treatment. For the co-inoculation of bacterial consortia, equal volumes (5 mL each) of two bacterial strains were combined to prepare the inoculum. Black gram seeds were surface sterilized as described previously and then treated with the specific bacterial combinations. Five seeds were sown equidistantly on the upper side of the sand in each jar. The lower portion of the glass bottle was filled with nitrogen-free nutrient medium, supplemented with 300 mM NaCl (w/v) to simulate saline conditions, as well as non-saline nutrient solution for control treatments. The nutrient solution was absorbed by the plants through capillary action facilitated by a cotton plug, following the modified Leonard jar method. The jars were regularly refilled with nutrient solution to maintain appropriate moisture levels and nutrient availability for plant growth and development. Black gram seedlings from each treatment were harvested 45 days after germination, carefully cleaned, and subjected to biometric measurements. These included root and shoot length, fresh weight of both root and shoot, as well as the fresh and dry weight of nodules. Additionally, the number of nodules per plant was recorded.

### 2.12 Effect of co-inoculation of bacterial isolates on black gram plant performance under salinity

Chlorophyll content was measured using an Apogee chlorophyll meter (MC-100), which records SPAD values to quantify the amount of light absorbed by chlorophyll in the leaf. The fourth leaf from the base to the tip of the plant was collected for this analysis. SPAD values were measured on 10 leaves per treatment, and the average value was calculated (Didal et al., 2022). Proline content was black gram determined according to the method outlined by Bates et al. (1973). Dried leaf samples were powdered and homogenized in aqueous sulfosalicylic acid. The mixture was then centrifuged at 8,000 rpm for 10 min, and the upper phase was collected. After adding ninhydrin and glacial acetic acid, the mixture was boiled for 30 min, and snap chilled in ice for 5 min. The reaction mixture obtained with an equal volume of toluene and absorbance was measured at 520 nm using a spectrophotometer. Nitrogen uptake was estimated using a KEL PLUS nitrogen analyzer (Pelican Instruments) following the Kjeldahl method, as modified by Biswas et al. (2020). In this process, 0.5 g of powdered plant material was digested in a di-acid mixture (9:1, H_2_SO_4_:HClO_4_) and filtered through Whatman No. 42 paper. The final volume was adjusted to 50 mL with distilled water. Boric acid solution was then titrated with 0.02 N H₂SO₂, and the blank titration was performed for accuracy. Nitrogen content in the plant material was calculated using the following formula:


$$N\;\text{in plant material}\;\left(\%\right)=\frac{0.02\times T\;\times \;0.014\times 50\times 50}{5\times 0.5}$$

(where, T = “Sample reading − Blank reading”).

To determine crude protein content, the following formula was used:


$$\text{Crude protien content}\;\left(\%\right)=\text{Nitrogen content}\;\left(\%\right)-6.25$$

The factor 6.25 converts nitrogen content to protein, assuming nitrogen is about 6% of protein. This ratio provides an estimate of protein content derived from nitrogen measurements.

### 2.13 Statistical analysis of experimented data

The collection of data is presented in form of average statistics, to evaluate the statistical differences of treatment means using a one-way analysis of variance (ANOVA). Duncan’s multiple range test was performed at *p* ≤ 0.05 for more in-depth comparisons. All statistical tests were conducted through using SPSS software (Version 16; IBM, Armonk, NY, United States).

## 3 Results and discussion

### 3.1 Preliminary soil analysis

In this study, the key soil parameters like pH, electrical conductivity (EC), availability of organic carbon, nitrogen, phosphorus, and potassium were analyzed through different standard methods. The obtained results depicted the soil pH from moderate to strongly alkaline. Mau soil had the lowest pH of 8.1, while Jaunpur and Azamgarh soils had the highest at 9.31 and 8.93, respectively.

In addition to pH, the EC varied in different soil samples, the soil of Azamgarh having the lowest mean value of moderate salty 7.79 dS m^−1^ and Jaunpur the highest at strong salty 9.61 dS m^−1^. Mau had an intermediate value of strongly salty 9.26 dS m^−1^. High soil salinity is often due to elevated chloride levels, contributing to higher EC values. The results were supported by previous studies in eastern Uttar Pradesh by Rai and Singh (2018).

Organic carbon content in the soil samples was found to range from 0.24 to 1.11%. Azamgarh had the lowest values (0.24–0.46%), while Jaunpur had the highest (0.63–1.11%). Low organic carbon levels suggest poor soil fertility.

Nitrogen content was obtained ranging from 103.22 to 276.73 kg/ha. The lowest values were in Azamgarh (103.22–189.16 kg/ha), and the highest in Jaunpur (166.82–276.73 kg/ha). Lower nitrogen availability is influenced by the semi-arid climate, which promotes rapid oxidation and reduces organic matter. Similar observations were found by Govindasamy et al. (2023).

The soil showed lower phosphorus content ranging from (79.03 to 114.27 kg/ha). Jaunpur had the lowest values (79.30–83.67 kg/ha), and Azamgarh had the highest (114.27 kg/ha). Higher phosphorus levels in many samples are likely due to the continuous application of phosphate fertilizers, which build up phosphorus despite their low efficiency and slow availability, as reported by Mandal and Sharma (2001). Additionally, low phosphorus soil observed in eastern Uttar Pradesh was reported by Tiwari et al. (2023).

However, the potassium content of the soil ranged from 99.68 to 138.88 kg/ha. Mau had the lowest values (99.68–105.61 kg/ha), while the highest was also in Jaunpur (137.76–138.88 kg/ha). High potassium content may be due to potash-rich minerals like micaceous and feldspar minerals in the rocks, as noted by Rai and Singh (2018). The physicochemical properties of soil samples from different location of eastern Uttar Pradesh is compiled in Table 1.

### 3.2 Isolation and halo-tolerant screening of bacterial isolates

**Figure 1:**
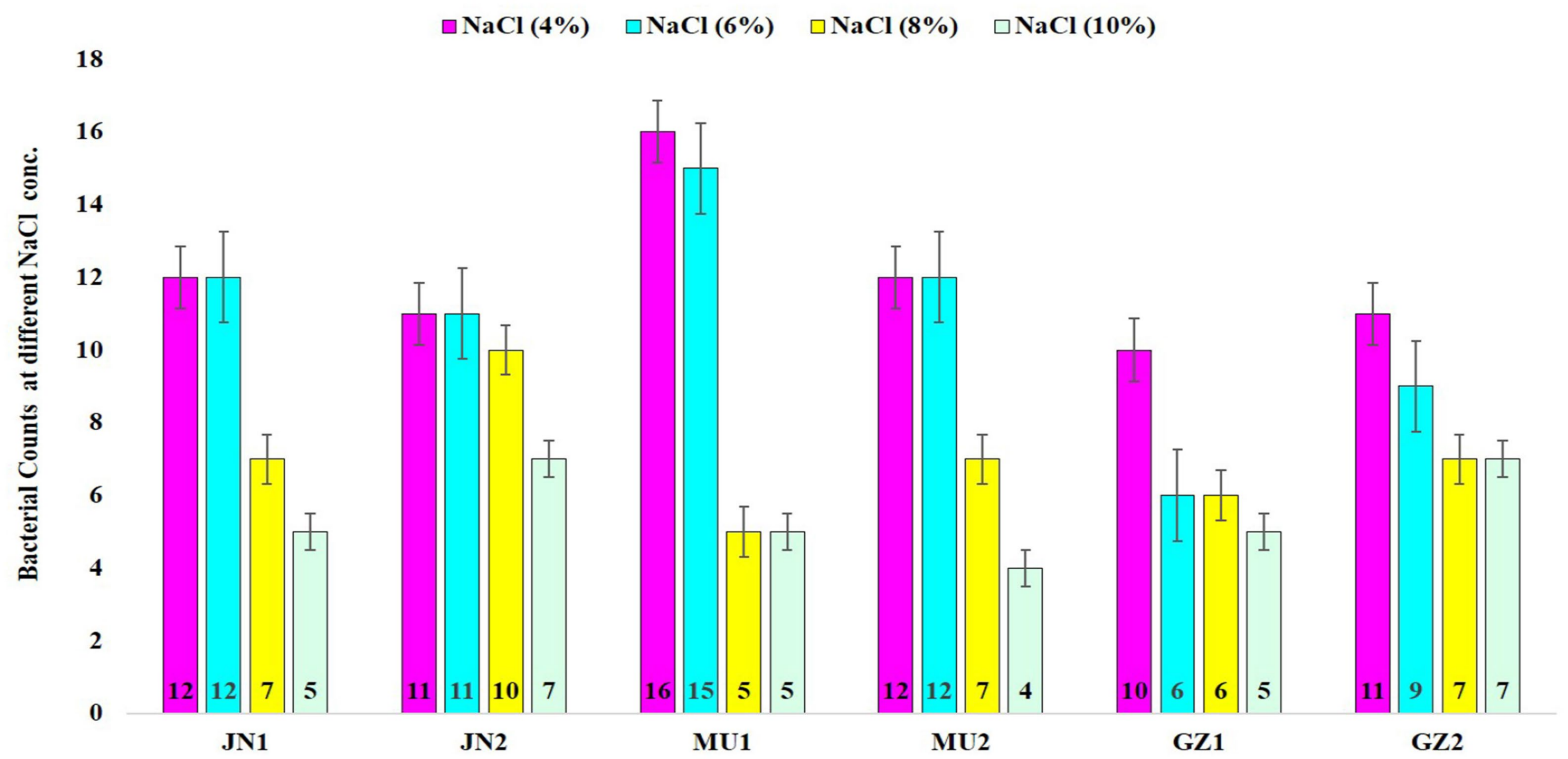
The graphical representation illustrates the tolerance of soil bacteria to salinity across different salt concentrations. JN1 and JN2, MU1 and MU2, and AZ1 and AZ2 correspond to the sampling locations in Jaunpur, Mau, and Azamgarh, respectively.

A total of 72 distinct bacterial strains were isolated from soil samples, along with an additional 28 strains isolated from the nodules of black gram. All the bacterial isolates were tested for salt stress under various NaCl concentrations: 4% (0.85 M), 6% (1.026 M), 8% (1.36 M), and 10% (1.71 M). The results showed that all isolates were able to tolerate NaCl at different concentrations, with 62.5% surviving at 6, 45.83% at 8, and 44.44% at 10% NaCl (Figure 1).

### 3.3 Determination of bacterial taxonomy and biochemical test

Biochemical tests play a vital role in bacterial identification and taxonomy. Based on biochemical and taxonomic characterization, 57.78% of bacterial isolates were found to be Gram’s-positive, spore-forming while 42.22% were Gram-negative, with a majority of rod-shaped. A total 34 four bacterial isolates were positive for amylase production, with 17 utilizing mannitol, 27utilizing dextrose, 13-lactose, and 21 positive for sucrose fermentation. In the MRVP test, nine cultures were positive for the methyl red test and four for the Voges Proskauer test. Nineteen cultures exhibited positive results in the nitrate reduction test and 22 were positive for the citrate utilization (Supplementary Figure S1 and Supplementary Table S2). In the presumptive tests for rhizobia, five colonies showed a whitish to pale pink color, indicating minimal dye absorption. Under microscopic examination, all isolates were observed as Gram-negative, rod-shaped, and non-spore-forming. These isolates did not grow on Hofer’s and glucose peptone agar medium and when treated with Benedict’s solution on ketolactose agar plates, they failed to produce yellow zone, indicating they were negative for the production of 3-ketolactose from lactose (Table 2).

**Table 2:** Taxonomic study and presumptive test for rhizobia.

Isolates	Steriomicrography	Gram staining	Endospore staining	Growth on glucose peptone agar	Congo red test	YEMA-BTB test	Hoffer’s alkaline test
PR1	Rod shaped	Negative	Non spore forming	No growth	Whitish to pale pink	Negative	No growth
PR3	Rod shaped	Negative	Non spore forming	No growth	Whitish to pale pink	Negative	No growth
PR4	Rod shaped	Negative	Non spore forming	Little growth	Whitish to pale pink	Negative	Little growth
PR6	Rod shaped	Negative	Non spore forming	No growth	Whitish to pale pink	Negative	Little growth
PR7	Rod shaped	Negative	Non spore forming	No growth	Whitish to pale pink	Negative	No growth

### 3.4 *In vitro* studies of compatibility test of salt-tolerant bacteria with nodule bacteria

Maximum salt-tolerant bacterial strains including SPR4, SPR11, SPR16, SPR17, and SPR20 showed compatibility with rhizobial strains PR1, PR3, PR4, PR6, and PR7. Notably, strainsPR1 and PR3 exhibited the highest compatibility with SPR11 and SPR16 (Supplementary Table S3). Similarly, Kumawat et al. (2024) reported that *Pseudomonas fluorescens* and *Enterococcus mundtii* show compatibility, enhancing growth characteristics, when applied consortium to mungbean compared to single inoculations. Additionally, Latha et al. (2009) isolated two *P. fluorescens* and *Bacillus subtilis* strains from tomato plants and found that all three strains were compatible for co-cultivation.

### 3.5 Determination of plant growth promotion activities and EPS production

In the assessment of ACC deaminase activity, SPR11 exhibited the maximum activity (48 μM mg^−1^ protein h^−1^) and PR7 was the least active (0.21 μM mg^−1^ protein h^−1^). Barnawal et al. (2014) reported an increase in ACC deaminase activity under 2% salt stress. The strain SPR11 exhibited the maximum phosphate solubilization ability, while PR4 showed the lowest. All bacterial isolates produced IAA ranging from 11.28 to 162.19 μg/mL. SPR11 strain showed the highest production (162.19) and SPR20 the lowest (11.28). The SPR11 strain also demonstrated the highest potassium solubilization efficiency in the presence of 2% NaCl. In a study, Saleem et al. (2021) also reported that salt-tolerant soil bacteria could produce up to 93 μg mL^−1^ of IAA. Khan et al. (2021) observed that mitigating effects by IAA synthesis through salt-tolerant bacterial isolates on soybean growth. Prittesh et al. (2020) reported that the bacteria DMBH2, NBH1, and G4PBH1 were the most efficient for phosphate solubilization and siderophore production at 2% concentration and improved seedlings and biomass of *Oryza sativa* in saline conditions.

SPR11 and SPR16 exhibited the maximum siderophore chelating efficiency. EPS production was recorded, ranging from 0.68 ± 0.11 to 2.38 ± 0.18 g/100 mL. The PR3 strain showed the highest capacity for EPS production, while SPR17 had the lowest. Table 3 represents the activity of plant growth promotion activities of different bacteria. Sharma et al. (2023) also reported the highest EPS level (2.82 g/100 mL) at a 4% NaCl concentration by the bacterial strain (CM94). The activity of plant growth-promoting (PGP) bacteria is summarized in Table 3.

**Table 3:** Plant growth-promoting attributes of different bacterial isolates at 2% NaCl.

Strain	IAA (μg/mL)	Siderophore production	ACC deaminase	Phosphate solubilization	Potassium solubilization	Zinc solubilization	EPS production
PR1	31.95 ± 2.38^de^	+	0.43 ± 1.08^de^	0.53 ± 0.08^de^	ND	ND	2.07 ± 0.38^a^
PR3	39.81 ± 1.42^d^	+	0.46 ± 1.12^de^	0.72 ± 0.07^de^	ND	1.2 ± 0.05^bc^	2.38 ± 0.18^a^
PR4	34.57 ± 2.56^e^	+	0.39 ± 1.27^de^	0.40 ± 0.05^de^	ND	1.1 ± 0.04^bc^	1.79 ± 0.14^ab^
PR6	32.16 ± 1.28^de^	+	0.32d ±1.52^de^	0.55 ± 0.02^de^	ND	ND	1.42 ± 0.17^ab^
PR7	33.74 ± 1.82^de^	+	0.21d ± 1.74^de^	ND	ND	ND	2.35 ± 0.16^a^
SPR4	40.22 ± 3.32^d^	+	17.66 ± 2.05^b^	0.89 ± 0.08^de^	ND	0.62 ± 0.07^d^	1.56 ± 0.9^ab^
SPR11	162.19 ± 3.45^a^	++	48.0 ± 3.76^a^	5.56 ± 0.1^a^	4.21 ± 0.18^a^	5.28 ± 0.06^a^	1.27 ± 0.16^ab^
SPR16	111.21 ± 4.62^b^	++	8.34 ± 1.29^c^	5.42 ± 0.12^a^	3.98 ± 0.12^ab^	3.59 ± 0.05^b^	0.79 ± 0.8^c^
SPR17	62.01 ± 2.85^c^	+	0.46 ± 1.11d^e^	2.28 ± 0.14^b^	1.89 ± 0.07^c^	1.58 ± 0.09^bc^	0.68 ± 0.11^bc^
SPR20	11.28 ± 1.96^f^	+	0.48 ± 1.34^d^	1.36 ± 0.11b^c^	0.89 ± 0.08^cd^	1.22 ± 0.11^bc^	0.83 ± 0.28^bc^

“+” indicates presence, “−” indicates absence. “+++”, high production; “++”, medium production; “+”, low production; “ND”, not detected; IAA, indole acetic acid; ACC, 1-aminocyclopropane-1-carboxylic acid; EPS, exopolysaccharides. Values are expressed as mean ± standard error, and the same letters indicate no significant differences at the 95% confidence level.

### 3.6 Analysis of ERIC and BOX PCR fingerprinting of salt-tolerant PGP bacteria and rhizobia

**Figure 2:**
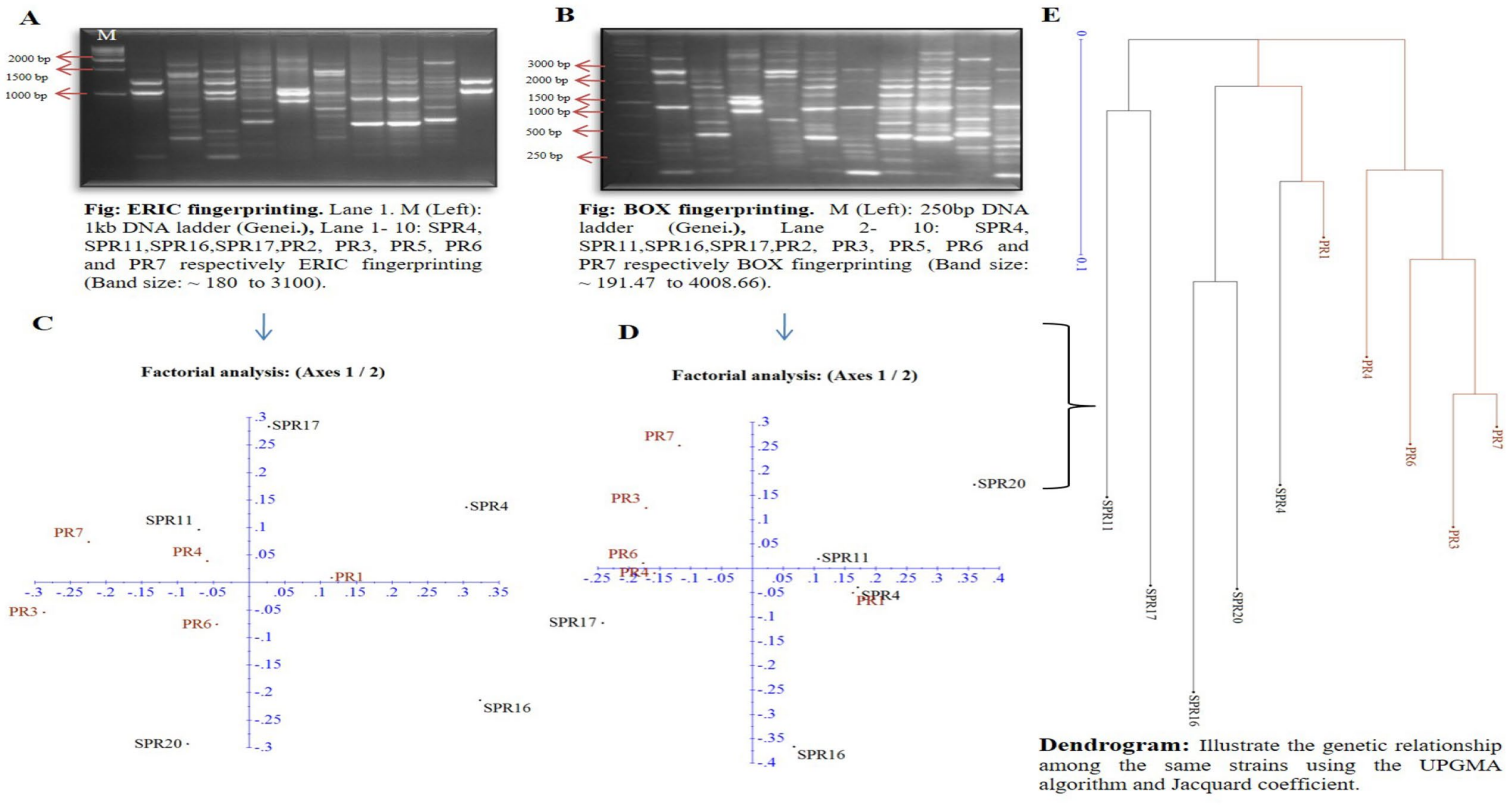
**(A,B)** Representing the band patterns obtained through gel electrophoresis, while **(C,D)** illustrate the factorial analysis and genetic relationship among 10 bacterial isolates based on a similarity matrix, generated from ERIC PCR and BOX PCR, respectively. The dendrogram in **(E)** represents the genetic relationships among the same bacterial strains using the UPGMA algorithm and Jaccard coefficient. All data were processed using DARWin statistical analysis (version 6.0.21).

Diagnostic techniques such as ERIC and BOX PCR were utilized to access the diversity of 10 selected bacterial isolates. The pattern of DNA fingerprinting patterns generated by ERIC PCR revealed 2 to 9 bands, while BOX PCR showed 2 to 12 bands (Figures 2A,B). A dendrogram constructed from the ERIC and BOX PCR profiles (Figure 2E) categorized all the isolates into four groups: group 1 consisted of isolates PR7, PR3, PR6, and PR4; group 2 included isolates PR1 and SPR4; group 3 comprised SPR20 and SPR16; and group 4 contained SPR17 and SPR11. The genetic individuality of the isolates was further confirmed through factorial analysis, revealing the genetic relationships among the 10 bacterial strains, based on the ERIC and BOX-PCR. The nodule bacteria were most distinct, located on the *x*-axis at distances of 0.11 (Figure 2C) and 0.25 (Figure 2D). In contrast, the salt-tolerant bacteria were positioned further along the negative *y*-axis at distances of 0.29 and 0.37, indicating a separation from the nodule bacteria. ERIC-PCR effectively helped a detailed analysis of genomic DNA differences among closely related strains. In contrast, BOX-PCR excelled at grouping strains by minimizing slight differences, which led to the formation of larger clusters. This suggests that different groups are genetically distinct from each other within the study. Previously, to study bacterial diversity researchers have also used ERIC and BOX PCR. These approaches have been widely used for detecting microbial ecology to differentiate genetic diversity at the intra-species level.

### 3.7 Molecular identification of salt tolerant PGP bacteria and rhizobia

**Figure 3:**
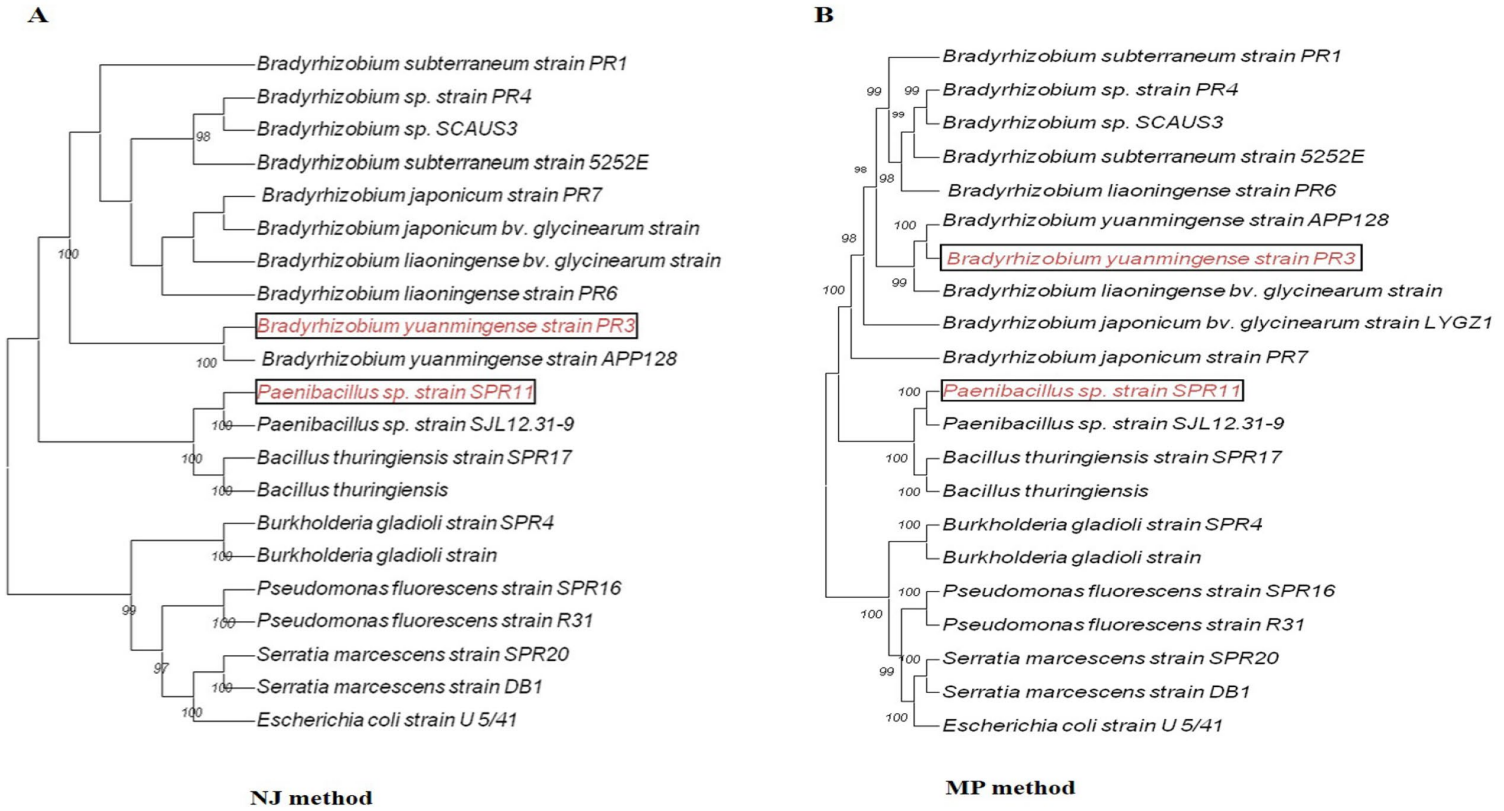
The phylogenetic relationship of different bacterial isolates with closely related strains, constructed based on the 16S rRNA gene sequences available from the NCBI database. The analysis was performed using neighbor-joining (NJ) and maximum parsimony (MP) methods in MEGA version 11 software, with *Escherichia coli* strain U5/41 used as the out-group.

The molecular identification of potent cultures was performed through phylogenetic analysis using full-length 16S rRNA gene sequences. BLAST searches were conducted to compare sequence similarities with closely related species using the EZbiocloud and NCBI databases. The identity of cultures was confirmed based on the phylogenetic tree constructed using neighbor-joining (NJ) and maximum parsimony (MP) methods (Figures 3A,B). The sequences of different cultures were submitted to the NCBI GenBank for accession (Supplementary Table S4). Additionally, the effective cultures were preserved at ICAR-NAIMCC in Mau, Uttar Pradesh, India, and assigned the accession numbers NAIMCC-B-02718 to NAIMCC-B-2727.

### 3.8 Co-inoculation effect on seed germination of black gram under salt (NaCl) supplements

**Figure 4:**
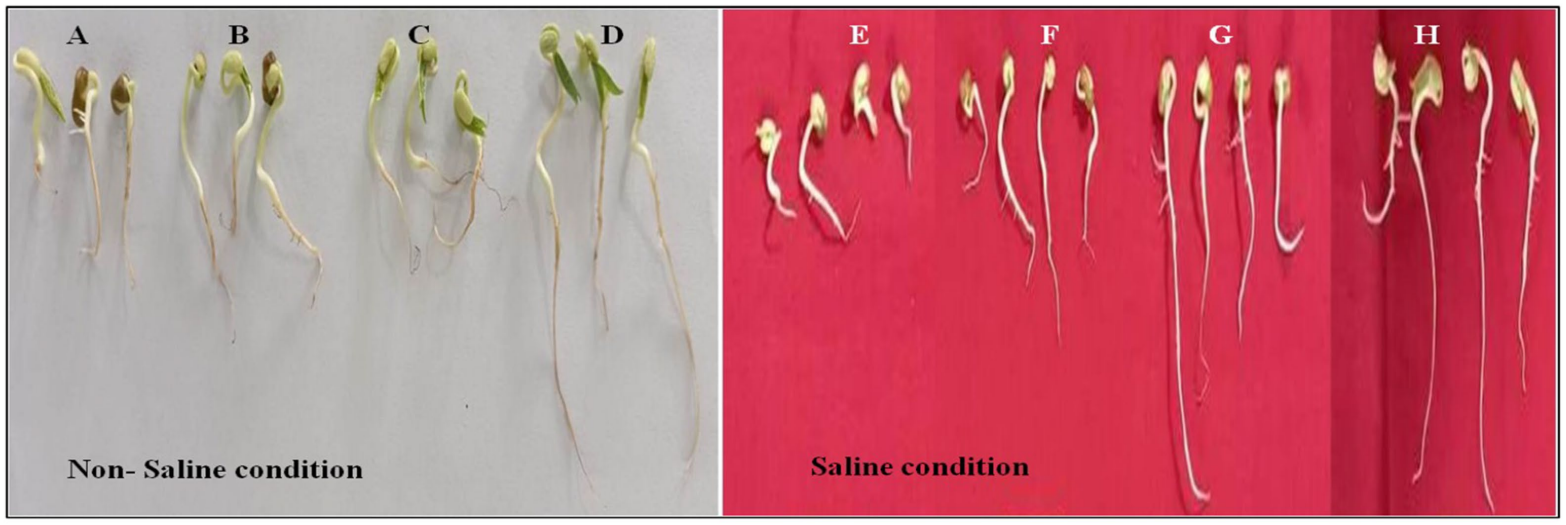
**(A–D)** Represents the effects of different treatments (untreated control, PR3, SPR11, and SPR11 + PR3) on seed germination in the absence of NaCl, with the untreated control serving as the negative control. **(E–H)** Show the effects of the same treatments (untreated control, PR3, SPR11, and SPR11 + PR3) on seed germination in the presence of 300 mM NaCl.

The seed germination assay conducted in this study assessed the effect of various bacterial treatments on seed performance under different saline conditions (200 mM, 300 mM NaCl, and without salt control). The seed germination data for 200 mM NaCl and without NaCl are shown in Supplementary Table S6. The different parameters such as pummel length, radicle length, shoot length, root length, and germination percentage were significantly affected by salt concentration. Across different bacterial treatments at 300 mM NaCl concentration was noted highest reduction in seed germination but co-inoculation of *Paenibacillus* sp. SPR11, *Bradyrhizobium yuanmingense* PR3 improved seed germination (Figure 4 and Table 4). Notably, co-inoculation of seeds with *B. yuanmingense* PR3 and *Paenibacillus* sp. SPR11 showed 40 and 45% higher seed germination percentages respectively, compared to the single rhizobial strains and the uninoculated control. Moreover, after 72 h under 300 mM saline conditions, shoot length reached 2.54 cm and root length measured 3.41 inches, showing significant enhancement compared to both single inoculations and the uninoculated control. These results suggest that specific bacterial combinations can improve seed performance in saline environments. Similar findings were published by Ilangumaran et al., 2021, who reported that *Rhizobium* sp. SL42 and other PGP bacterial treatments increased soybean germination rates at 100 and 125 mM NaCl, though no significant enhancement was seen at higher salt concentrations (175 and 200 mM NaCl).

**Table 4:** Effect of various treatments on seed germination of black gram under saline and non-saline conditions.

Salt level	Treatments	Initiation time (h)		Germination (%)	RL (inch)		SL (cm)	
		RG	PG		48 h	72 h	48 h	72 h
Non-saline conditions	PR3	24	48	95%	1.13^c^	2.20^c^	1.02^b^	1.80^c^
	SPR11	24	48	100%	1.75^b^	2.87^b^	1.70^a^	2.67^b^
	SPR11 + PR3	24	48	100%	2.47^a^	3.65^a^	1.87^a^	2.87^a^
	Control	24	48	95%	1.05^c^	1.67^d^	1.025^b^	1.57^d^
Saline-condition (300 mM NaCl)	PR3	24	72	60%	0.25^c^	0.53^c^	0.034^c^	0.25^c^
	SPR11	24	48	90%	1.4^b^	1.92^b^	1.16^b^	1.80^b^
	SPR11 + PR3	24	48	95%	2.4^a^	3.41^a^	1.84^a^	2.54^a^
	Control	24	72	50%	0.12^c^	0.34^c^	0.000^d^	0.14^c^

Impact of various treatments under saline and non-saline (300 mM) conditions on SL, shoots length; SFW, shoot fresh weight; RL, root length; RFW, root fresh weight; NC, nodule counts; NFW, nodule fresh weight and NDW, nodule dry weight per plant. The table data represent as means with standard deviations (*n* = 5). Different letters indicate significant differences between treatments, as determined by Duncan’s multiple range test (*p* < 0.05).

### 3.9 Co-inoculation effect on nodulation and growth of black gram under N-free-saline conditions

In a Leonard jar experiment, statistical analysis was conducted on agronomic factors after a 45-day seedling period dual inoculation with strains PR3 and SPR11 significantly enhanced several plant growth parameters. Specifically, increased root length (47.71 and 50.35%) root weight (57.50 and 62.92%) shoot length (37.94 and 49.49%), shoot weight (41.73 and 47.83%) and improved root architectures compared to single rhizobial inoculation and the uninoculated control. Additionally, co-inoculation of strains PR3 and SPR11 significantly increased nodule numbers (75.74%), fresh nodule weight (75.65%), and dry nodule weight (80.28%) compared to the single rhizobial treatment (Table 5). These finding align with work of Gopalakrishnan et al. (2022), who reported that salt tolerant plant growth-promoting bacteria *Paenibacillus dendritiformis* in bacteria substantially induced root (78–102%) and shoot weights (37–57%) in three melon varieties treated with saline water. Additionally, Egamberdieva et al. (2017) found that combination of *Bradyrhizobium japonicum* USDA 110 with the TSAU1 strain enhanced salt tolerance and growth in soybean by improving protein, nitrogen and phosphorus as well as root architecture under hydroponic conditions. Another study supports our findings with Korir et al. (2017), who observed abundant nodulation in common beans with the use of *Bacillus megaterium* PAU 983 C and *Paenibacillus polymyxa* IITA-PAU 983 C together.

**Table 5:** Effect of strain PR3 and SPR11 on black gram growth under NaCl (300 mM) stressed condition.

Treatment	SW (cm)	SFW (mg)	RL (cm)	RFW (mg)	NC (mg)	NFW (mg)	NDW (mg)
PR3	26.40^c^	293.44^b^	19.68^c^	124.93^c^	14.00^b^	74.66^b^	6.18^b^
SPR11	38.34^b^	477.70^a^	29.33^b^	253.70^b^	0.00^c^	0.000^c^	0.00^c^
PR3 + SPR11	42.54^a^	503.66^a^	37.71^a^	294.00^a^	51.71^a^	306.70^a^	31.34^a^
Control	23.00^d^	262.71^b^	18.72^c^	109.60^c^	0.00^c^	0.00^c^	0.00^c^

Impact of various treatments under saline (300 mM) conditions on SL, shoots length; SFW, shoot fresh weight; RL, root length; RFW, root fresh weight; NC, nodule counts; NFW, nodule fresh weight and NDW, nodule dry weight per plant. The table data are presented as means with standard deviations (*n* = 5). Different letters indicate significant differences between treatments, as determined by Duncan’s multiple range test (*p* < 0.05).

### 3.10 Effect of co-inoculation on chlorophyll pigments and osmolytes

**Figure 5:**
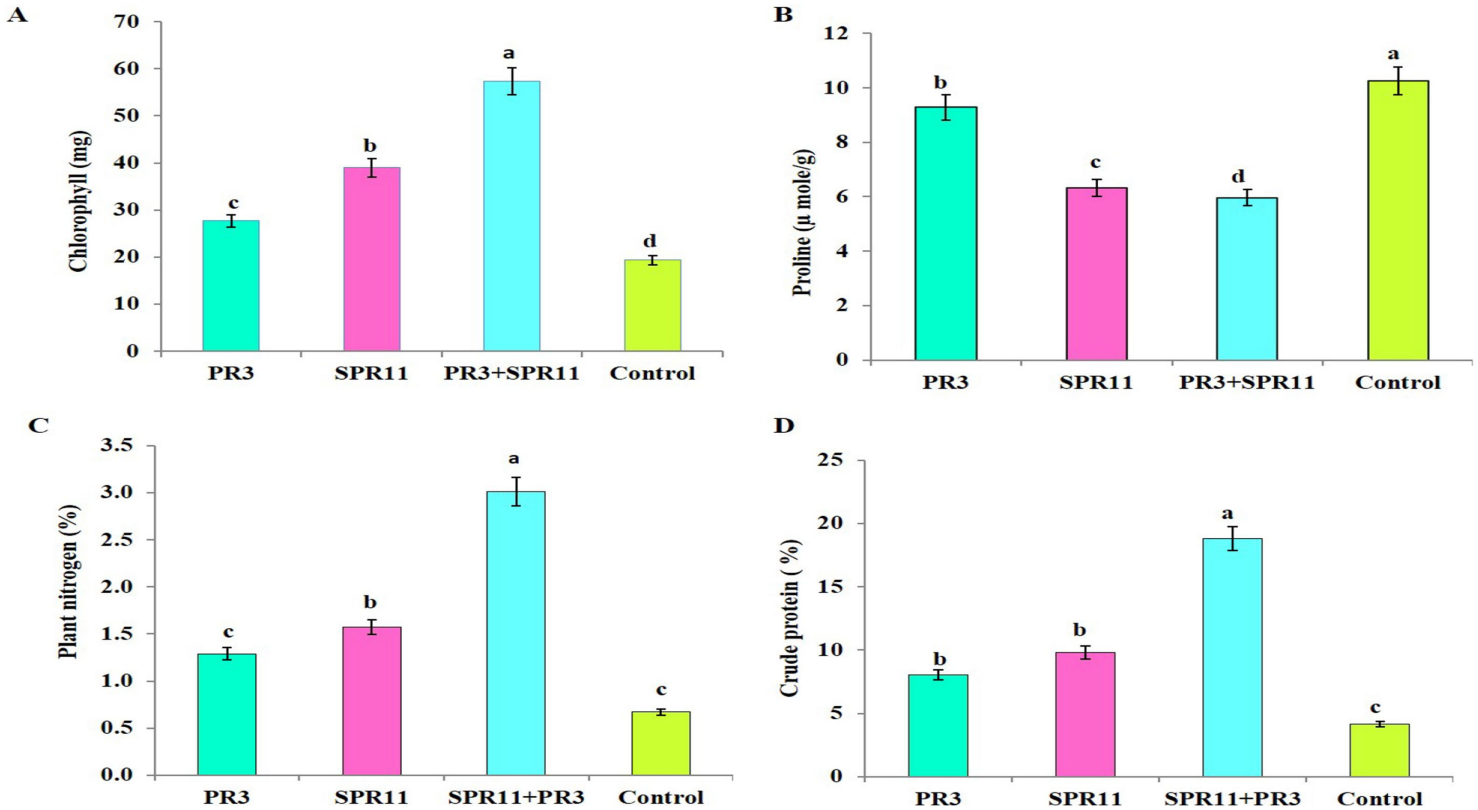
The effect of various treatments on **(A)** chlorophyll content (SPAD value), **(B)** proline content, **(C)** nitrogen uptake, and **(D)** crude protein is shown. The data are presented as the means of four independent experiments ± standard error (SE), with *N* = 20 (4 treatments × 5 replicates). Different letters indicate statistically significant differences (*p* < 0.05) as determined by Duncan’s multiple range test.

Chlorophyll pigments were significantly varied due to 300 mM NaCl salt stress across different treatments. Co-inoculation with *Paenibacillus* sp. SPR11 and the rhizobial strain *Bradyrhizobium yuanmingense* PR3 resulted in increases of 31.58, 51.74, and 66.28% in chlorophyll content compared to the single inoculations of SPR11 and PR3, as well as the uninoculated control (Figure 5A). Bhise et al. (2016), was reported that co-inoculation of rhizobia and *Enterobacter cloacae* Strain KBPD able to promoted the mung bean growth and increased chlorophyll content under varying NaCl concentrations of (50 to 150 mM).

In response to salinity stress, plants produce osmolytes, especially proline, to overcome the adverse effects. Our results indicate that plants inoculated with *Paenibacillus* sp. SPR11 and *Bradyrhizobium yuanmingense* PR3 significantly reduced proline content under 300 mM NaCl salt stress by 5.53, 35.66, and 41.81%, respectively compared to single inoculation of SPR11 and PR3 as well as the uninoculated control (Figure 5B). Gupta and Pandey (2020) reported that ACC deaminase-producing strains *Aneurinibacillus aneurinilyticus* strain AIOA1 (MH645748) and *Paenibacillus* sp. strain SG_AIOA2 (MH645749) reduced proline content common be an under 100 mM saline condition. Additionally, Bisht et al. (2019) reported that *Paenibacillus lentimorbus* NRRL B-30488 exhibiting plant growth-promoting traits significant reduced proline content by 30% in chickpea plant compared to the uninoculated control.

### 3.11 Uptake of nitrogen content and enhanced crude protein in black gram

The nutrient content in black gram varied significantly across different treatments. In this study, the co-inoculation of *Paenibacillus* sp. SPR11 and *Bradyrhizobium yuanmingense* PR3 showed the highest nitrogen uptake and crude protein levels, showing increases 57.15, 47.84, and 77.74% compared to single inoculations PR3, SPR11 and the uninoculated control (Figures 5C,D). These findings align with Jabborova et al. (2021), who observed that single inoculation of *Bradyrhizobium japonicum* USDA 110 increased N content by 29 and 28%, while co-inoculation of *Bradyrhizobium japonicum* USDA 110 and *Pseudomonas putida* NNU8 resulted in a significant improvement of nitrogen content by 44 and 35% in soybean. Additionally, PGPR combined with *Rhizobium* strain AR-2–2 k produced more nodules, a greater dry weight of nodules, and higher nitrogen content in pigeon pea shoots compared to single inoculation and uninoculated control in pigeon pea reported by Tilak et al. (2006), according to Fitouri et al. (2011) total nitrogen are important indicators that emphasized that shoot dry weight and of strain effectiveness.

## 4 Conclusion

The persistent global population growth has led to an increased demand for food, thereby intensifying the need for enhanced agricultural productivity. Among the numerous constraints to achieving improved crop yields and quality, soil salinity remains a major limiting factor. Traditional strategies to address salinity-related stress are often costly and environmentally burdensome. However, more cost-effective and sustainable approaches, such as the application of microbes exhibiting plant growth-promoting rhizobacteria (PGPR) activity, have shown promise in improving plant growth, accelerating seed germination, enhancing seedling emergence, and providing protection against abiotic stressors. In the present study, six soil samples from saline regions of eastern Uttar Pradesh, India were screened for salt-tolerant bacterial strains. The bacterial strain SPR11, isolated from a salt-affected site in Mau, demonstrated significant plant growth-promoting and salt stress-mitigating properties, particularly when co-inoculated with the nodule-forming strain PR3. Molecular identification through sequencing revealed that SPR11 and PR3 were genetically identical to *Paenibacillus* sp. and *Bradyrhizobium yuanmingense*, respectively. In both seed germination and greenhouse assays, SPR11 exhibited superior performance in alleviating salt stress compared to the single rhizobia PR3, particularly in black gram (*Vigna mungo*). The growth-promoting effects of SPR11 were attributed to its ability to produce phytohormones, exopolysaccharides (EPS), osmolytes, and other bioactive compounds that mitigate salt-induced stress. Additionally, SPR11 enhanced plant growth and nodulation through mechanisms such as the production of 1-aminocyclopropane-1-carboxylate (ACC) deaminase, indole-3-acetic acid (IAA), siderophores, and the solubilization of key nutrients, including phosphorus, potassium, and zinc. Furthermore, SPR11 contributed to improved plant defense mechanisms by increasing chlorophyll content, enhancing nitrogen uptake, boosting crude protein levels, and reducing proline accumulation an indicator of osmotic stress thereby demonstrating its potential to alleviate the phytotoxic effects of salinity. These results suggest that SPR11 may serve as a cost-effective and environmentally sustainable biotechnological tool for managing saline soils and improving crop productivity, particularly in salt-affected regions.

## Data Availability

The original contributions presented in the study are included in the article/Supplementary material, further inquiries can be directed to the corresponding authors.
