# Supplementary material for: Stress-relieving plant growth-promoting bacterial co-inoculation enhances nodulation and nitrogen uptake in black gram under nitrogen-free saline conditions
Source: Front Microbiol. 2025 Jan 3;15:1516748. doi: 10.3389/fmicb.2024.1516748 (PMC11739075; doi:10.3389/fmicb.2024.1516748)
Supplement: Supplementary file 1 [file Supplementary_file_1.docx]

**Supplementary Table 1. Details of soil collection sites and their physico-chemical properties.**

| **Collection Sites** | **Latitude** | **Longitude** | **Sample type** | **Soil texture** |
| --- | --- | --- | --- | --- |
| JN1 | 25.967100 | 82.682800 | Soil | Loamy |
| JN1 | 25.754900 | 82.687060 | Soil | Loamy |
| MU1 | 25.972970 | 82.749210 | Soil | Loamy with white crust |
| MU1 | 25.974820 | 83.574640 | Soil | Loamy with white crust |
| AG1 | 26.055200 | 82.681500 | Soil | Loamy |
| AG1 | 25.972969 | 82.749207 | Soil | Loamy |
| NMU | 25.972970 | 82.749210 | Root/nodules | - |
| NGZ | 25.974820 | 83.574640 | Root/nodules | - |

**Note:** The soil sampling sites are designated as follows: JN for Jaunpur, AG for Azamgarh and MU for Mau. Data are presented as collection site, location, soil type and soil texture.

**Supplementary Table 2:** Morphological and biochemical characterization of soil and nodules bacteria

| **Isolates code** | **Isolation media and Morphology** | | | | **G** | **Sugar Fermentation** | | | | **MR** | **VP** | **NR** | **CU** | **A** |
| --- | --- | --- | --- | --- | --- | --- | --- | --- | --- | --- | --- | --- | --- | --- |
|  | **Media** | **Shape** | **Motility** | **Endospore** |  | **M** | **D** | **L** | **S** |  |  |  |  |  |
| 1 | SCA | Rod- shaped | Motility | + | + | - | + | - | + | - | - | + | - | + |
| 2 | NA | Rod- shaped | Motile | + | + | - | + | - | - | + | - | + | + | - |
| 3 | NA | Rod -shaped | Motile | + | + | - | + | + | + | - | - | + | - | + |
| SPR4 | NA | straight rods | Motile | - | - | - | + | - | - | + | + | + | + | + |
| 5 | NA | Rod shaped | Motile | + | + | - | + | + | - | - | - | + | - | + |
| 6 | NA | Rod shaped | Motile | + | + | - | + | - | - | - | - | - | + | - |
| 7 | SCA | Rod shaped | Motile | + | + | - | + | - | + | - | - | + | - | + |
| 8 | KB | Rod shaped | Motile | + | - | - | + | - | + | - | - | - | + | + |
| 9 | NA | Rod shaped | Motile | + | + | + | + | + | - | + | - | - | + | + |
| 10 | NA | Rod shaped | Motile | + | - | - | - | - | - | - | - | - | - | - |
| SPR 11 | NA | Straight rod | Motile | + | + | - | + | - | + | - | - | + | + | + |
| 12 | NA | Rod shaped | Motile | + | + | - | - | - | + | - | - | - | + | + |
| 13 | NA | Rod shaped | Non-motile | + | + | + | + | - | + | + | - | + | - | + |
| 14 | NA | Rod shaped | Motile | + | + | - | - | - | - | - | - | + | - | - |
| 15 | SCA | Rod shaped | Non-motile | + | + | + | + | + | + | + | + | - | - | + |
| SPR 16 | KB | Rod shaped | Motile | - | - | - | + | - | + | - | - | + | - | + |
| SPR 17 | NA | straight rods | Motile | + | + | - | + | - | + | - | - | + | - | - |
| 18 | SCA | Rod shaped | Motile | + | + | - | - | - | - | - | - | + | - | + |
| 19 | SCA | Rod shaped | Motile | + | + | - | + | + | + | - | - | + | - | + |
| SPR 20 | NA | Rod shaped | Motile | - | - | - | + | + | - | + | + | + | + | - |
| 21 | KB | Rod shaped | Non-motile | + | + | - | + | + | + | - | - | + | - | + |
| 22 | NA | straight rods | Motile | + | + | - | + | - | + | - | - | + | + | + |
| 23 | NA | Rod shaped | Motile | + | + | - | + | - | - | + | - | - | - | - |
| 24 | R2A | Rod shaped | Motile | + | - | - | + | - | - | - | - | + | - | + |
| 25 | SCA | Rod shaped | Non-motile | - | + | - | + | - | - | - | - | - | - | + |
| 26 | SCA | Rod shaped | Motile | - | + | + | + | + | + | - | - | - | - | - |
| 27 | R2A | straight rods | Motile | - | - | - | + | - | - | - | - | - | + | + |
| 28 | NA | Rod shaped | Motile | - | - | - | + | - | - | - | - | + | - | + |
| 29 | KB | Rod shaped | Motile | + | + | - | + | - | - | - | - | - | - | + |
| 30 | R2A | Rod shaped | Motile | + | + | - | + | - | - | + | - | - | + | - |
| 31 | R2A | straight rods | Non-motile | + | + | - | - | + | - | - | - | - | - | + |
| 32 | NA | Rod shaped | Non-motile | + | + | - | - | + | + | + | + | - | + | + |
| PR1 | SCA | Rod shaped | Non-motile | - | - | + | - | + | + | - | - | - | + | + |
| PR2 | NA | Rod shaped | Motile | - | - | + | - | - | + | - | - | - | - | + |
| PR4 | NA | Rod shaped | Motile | - | - | + | - | - | - | - | - | - | + | + |
| PR5 | NA | Rod shaped | Motile | - | - | + | - | - | - | - | - | - | + | + |
| PR6 | NA | Rod shaped | Motile | - | - | + | - | - | - | - | - | - | + | + |
| PR7 | NA | straight rods | Motile | - | - | + | - | + | + | - | - | + | - | - |
| PR8 | SCA | Rod shaped | Motile | - | - | + | + | - | - | - | - | - | - | + |
| PR9 | KB | Rod shaped | Motile | - | - | + | - |  | - | - | - | - | + | + |
| PR10 | NA | Rod shaped | Motile | - | - | + | - | - | - | - | - | - | + | + |
| PR11 | NA | Rod shaped | Motile | - | - | + | - | - | - | - | - | - | + | - |
| PR12 | NA | straight rods | Motile | - | - | + | - | + | + | - | - | - | + | + |
| PR13 | NA | Rod shaped | Motile | - | - | + | - | - | + | - | - | - | + | + |
| PR14 | NA | Rod shaped | Motile | - | - | + | - | - | + | - | - | - | + | + |

*****assigned codes: Gram’s staining – G, Sugar fermentation test (M, Mannitol, Dextrose – D, Lactose – L, Sucrose – S), Methyl red test – MR, Voges Prousker test – VP, Nitrate reduction test – NR, Citrate utilization test – CU and Amylase production test – A.

**Supplementary Table 3. In vitro results of compatibility test of salt tolerant bacteria with nodule bacteria at 2% of (NaCl).**

| **Pairs of isolates** | **Interaction** | **Zone of Inhibition (mm)** | **Compatibility** |
| --- | --- | --- | --- |
| PR1+SPR20 | ++ | - | Incompatible |
| PR1+SPR11 | +++ | - | compatible |
| PR1+SPR16 | +++ | - | Incompatible |
| PR1+SPR17 | + | - | Incompatible |
| PR1+SPR4 | + | - | compatible |
| PR3+SPR20 | ++ | - | In compatible |
| PR3+SPR11 | +++ | - | compatible |
| PR3+SPR16 | +++ | - | compatible |
| PR3+SPR17 | + | - | compatible |
| PR3+SPR20 | - | + | In compatible |
| PR4+SPR20 | ++ | - | In compatible |
| PR4+SPR11 | + | - | compatible |
| PR4+SPR16 | + | - | compatible |
| PR4+SPR17 | + | - | compatible |
| PR4+SPR20 | - | + | In compatible |
| PR4+SPR4 | + | - | Compatible |
| PR6+SPR20 | + | + | Incompatible |
| PR6+SPR11 | + | - | compatible |
| PR6+SPR16 | + | - | compatible |
| PR6+SPR17 | - | + | Incompatible |
| PR6+SPR4 | + | - | Compatible |
| PR7+SPR20 | + | - | compatible |
| PR7+SPR11 | + | - | compatible |
| PR7+SPR16 | - | + | Incompatible |
| PR7+SPR17 | + | - | compatible |
| PR7+SPR4 | + | - | Compatible |

Minimum **(+)**, medium (++) and maximum (**+++)** Indicates no inhibition or positive interaction, **-**: Indicates inhibition or negative interaction strains

**Supplementary Table 4. Molecular identification of bacterial isolates based on 16S rRNA gene sequencing.**

| Sequence ID | Blast-related sequence | Accession | E value | Homology |
| --- | --- | --- | --- | --- |
| PR3 | *Bradyrhizobium yuanmingense* | MT605283.1 | 0.0 | 99% |
| PR6 | *Bradyrhizobium liaoningense* | MT605285.1 | 0.0 | 99% |
| PR7 | *Bradyrhizobium japonicum PR7* | MT605286.1 | 0.0 | 99% |
| PR1 | *Bradyrhizobium subterraneum PR1* | MT605281.1 | 0.0 | 99% |
| PR4 | *Bradyrhizobium* sp. | MT605282.1 | 0.0 | 98% |
| SPR4 | *Burkholderia gladioli* | OK481386 | 0.0 | 100% |
| SPR11 | *Paenibacillus* sp. | OK481384 | 0.0 | 100% |
| SPR16 | *Pseudomonas fluorescens* | OK481388 | 0.0 | 99% |
| SPR17 | *Bacillus thuringiensis* | OK481387 | 0.0 | 100% |
| SPR20 | *Serratia marcescens* | OK481385 | 0.0 | 99% |

| **Nitrogen free media for seed germination and plant growth** | |
| --- | --- |
| **Chemicals** | **Weight for 1liter of media** |
| CaHPO4- | 1g |
| MgSO4.7H2O | 0.2 |
| FeCl2-.2g | 0.2 |
| K2HPO4 | 0.2 |
| NaCl- | 0.2g |
| **Trace element** |  |
| Boric acid | 0.05% |
| MnSO₄H₂O | 0.05% |
| ZnSO4 | 0.005% |
| Na2MoO4 -0.005% | 0.005% |
| CuSO4.7H2O-0.002% | 0.002% |
|  |  |

**Supplementary Table 5:** Nitrogen-fee media using in seed germination and green house evaluation.

**Table 6. Effect of various treatments on seed germination of black gram under saline (200mM NaCl) and without salt condition**

| **Non saline** | **Treatments** | **Initiation time (Hrs.)**  **RG PG** | | **Germination (%)** | **Root length (cm)**  **Time interval**  **48hrs 72hrs** | | **Shoot length(cm)**  **Time interval**  **48hrs 72hrs** | |
| --- | --- | --- | --- | --- | --- | --- | --- | --- |
| Non- saline conditions | PR3 | 24 | 48 | 100% | 1.66e | 2.17e | 1.72c | 2.91e |
|  | PR4 | 24 | 48 | 100% | 1.67e | 2.38c | 1.36d | 2.89b |
|  | SPR16+PR3 | 24 | 48 | 100% | 2.16c | 3.11b | 1.68bc | 2.23bc |
|  | SPR11+PR3 | 24 | 48 | 100% | 3.98a | 5.40a | 2.16a | 4.38a |
|  | SPR17+PR4 | 24 | 48 | 100% | 2.68b | 3.45b | 1.87b | 2.67b |
|  | SPR4+PR4 | 24 | 48 | 100% | 2.26c | 2.50c | 1.86b | 2.79b |
|  | SPR20+PR4 | 24 | 48 | 100% | 1.97d | 2.24c | 1.69bc | 2.52bc |
|  | Control | 24 | 48 | 100% | 1.59e | 2.0cd | 1.14e | 2.10bc |
| 200mM | PR3 | 24 | 72 | 70% | 0.46d | 0.93f | 0.64d | 0.95e |
|  | PR4 | 48 | 72 | 60% | 0.33d | 0.81f | 0.37d | 0.66e |
|  | SPR16+PR3 | 24 | 72 | 95% | 1.54b | 2.46c | 0.98c | 1.63c |
|  | SPR11+PR3 | 24 | 48 | 100% | 2.68a | 3.82a | 2.43a | 3.26 a |
|  | SPR17+PR4 | 24 | 48 | 100% | 1.82b | 2.92b | 1.69b | 2.47b |
|  | SPR4+PR4 | 24 | 72 | 95% | 1.23bc | 1.56e | 0.64d | 1.05d |
|  | SPR20+PR4 | 24 | 48 | 90% | 1.26bc | 1.90d | 0.76d | 1.19d |
|  | Control | 24 | 72 | 50% | 0.22d | 0.64e | 0.000e | 0.36f |

**Note:** Impact of various treatments under saline (200 mM) and without salt on SL: Shoots length; RL, Root length; RG, Radical growth; PG, Plumule growth at different time interval). The table data represent as means with standard deviations (n =4 Different letters indicate significant differences between treatments, as determined by Duncan’s multiple range test (p ≤ 0.05).

**Supplementary Figure1: taxonomic and biochemical characterization of salt tolerant and nodule bacteria**


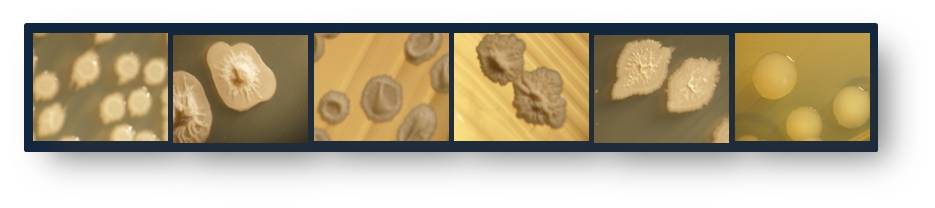

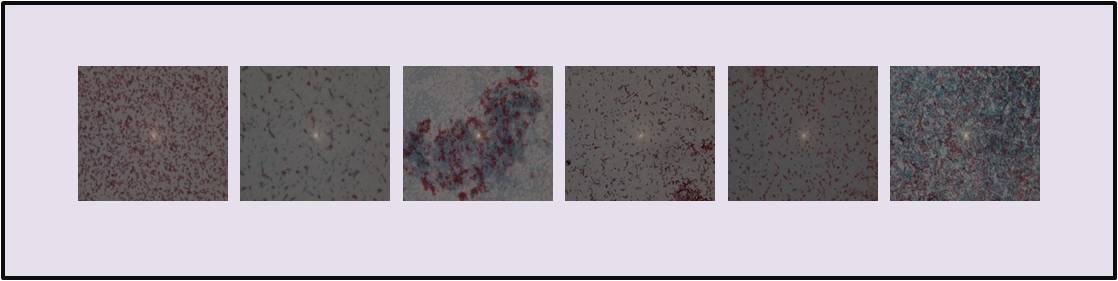

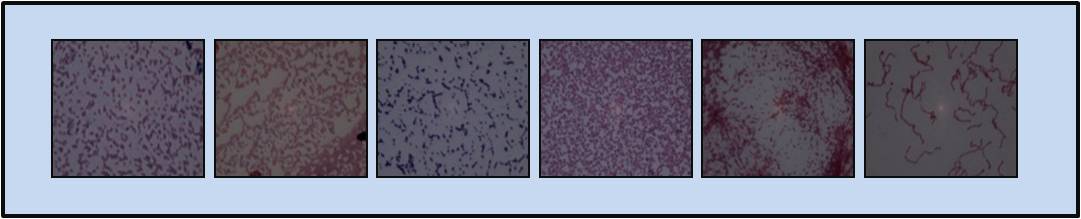

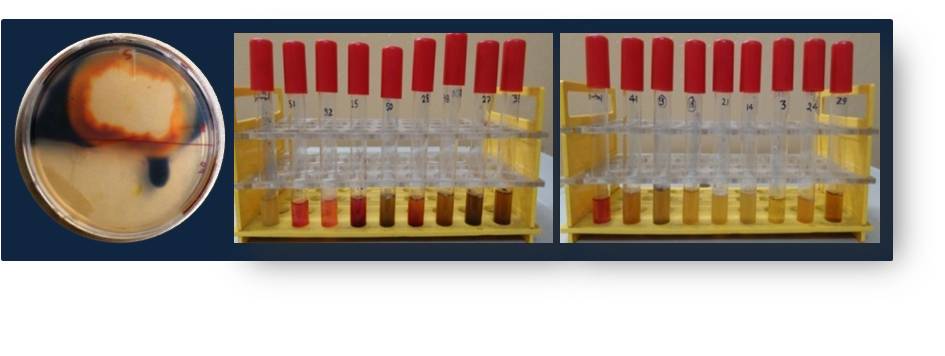


**A**

**B**

**C**

D

**Note:** Figure showed A- steriomicrograph B-Gram staining, C – endospore staining D- biochemical test
